# Facile Fabrication of Anthocyanin-Nanocellulose Hydrogel Indicator Label for Intelligent Evaluation of Minced Pork Freshness

**DOI:** 10.3390/foods12132602

**Published:** 2023-07-05

**Authors:** Xiangyong Meng, Qinqin Shen, Teng Song, Honglei Zhao, Yong Zhang, Aiqing Ren, Wenbin Yang

**Affiliations:** 1 Anhui Provincial Key Laboratory of Molecular Enzymology and Mechanism of Major Diseases, Key Laboratory of Biomedicine in Gene Diseases and Health of Anhui Higher Education Institutes, College of Life Sciences, Anhui Normal University, Wuhu 241000, China; 2 School of Ecology and Environment, Anhui Normal University, Wuhu 241000, China; 3 Weifang Inspection and Testing Center, Weifang 261100, China; 4 Institute of Food Research, Hezhou University, Hezhou 542899, China

**Keywords:** nanocellulose, anthocyanin, hydrogel, intelligent indicator label, freshness

## Abstract

In order to develop a reliable and rapid method for meat freshness detection, nanocellulose (TOCNF) prepared via the TEMPO (2,2,6,6-tetramethylpiperidine oxidation) oxidation method was used as raw material to prepare hydrogels using Zn^2+^ coordination and binding. Physicochemical properties such as water absorption and porosity were analyzed. It was further used to select suitable hydrogels for the preparation of indication labels after anthocyanin adsorption, and it was applied in the freshness detection of fresh minced pork. Five percent TOCNF (*w*/*w*) aqueous solution was homogenized by high shear for 4 min, and 20% (*w*/*w*) zinc chloride solution was added to it, so that the concentration of zinc ions could reach 0.25 mol/L. After standing for 24 h, the hydrogel was obtained with good water absorption and a porous three-dimensional network structure. The activation energies of volatile base nitrogen (TVBN) and anthocyanin indicating label color changes were 59.231 kJ/mol and 69.453 kJ/mol, respectively. The difference between the two is within 25 kJ/mol, so the prepared indicator label can accurately visualize the shelf life of fresh pork.

## 1. Introduction

With the improvement of the quality of life, people’s attention to food quality and safety is increasing, and the demand for fast and effective access to food quality and freshness is also increasing [1]. To date, researchers have developed a variety of intelligent food indicator labels to monitor the status changes in packaged products, such as food freshness, spoilage and maturity, microbial counts, and product quality information such as texture and flavor [2]. The freshness indicator label uses target chemical substances such as carbon dioxide [3] or volatile nitrogen compounds [4] to dissolve in water to cause a change in pH value, thereby causing a color change on the indicator label. The color change in the label is highly sensitive and effective to humidity, but excessive humidity will cause the label to absorb water, swell, and become damaged [5]. Therefore, the hydrophilicity and stability of the material need to be considered in the preparation of the freshness indicator label. Therefore, the hydrophilicity and stability of materials are the prerequisites for the successful preparation of freshness indicator labels. Studies [6] have shown that in addition to chemical dyes such as bromothymol blue and methyl red, natural pH-sensitive dyes [2,6] such as anthocyanin, curcumin, and madder extract [7] were used to interact with highly hydrophilic chemical substances such as starch, chitosan, polyvinyl alcohol, and other substances to prepare food freshness indicator labels which have good indication effects and natural pigments with low toxicity, and the indicator labels must not pollute the food.

Hydrogel is a kind of material with a stable three-dimensional network structure made of natural or synthetic hydrophilic substances by means of thermal gelation, ionic interaction, chemical bonding or cross-linking, and it has the ability to hold a large amount of water [8]. If highly hydrophilic materials such as hydrogels are used to prepare indicator labels, the adverse effects of humidity on the sensitivity and effectiveness of color changes can be avoided, and the stability of the color response of pH-sensitive dyes to target chemicals can be improved [9]. Among them, the advantages of natural polymer-based hydrogels such as starch and cellulose compared with chemically synthesized materials in terms of biodegradability and biocompatibility have attracted more and more attention [9,10].

**Figure 1:**
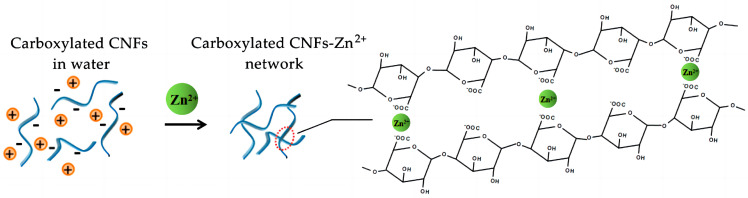
Schematic diagram of Zn^2+^ cross-linked with TOCNF for gelation.

Oxidized nanocellulose (TOCNF), which is obtained by oxidizing the hydroxyl group at the C6 position of cellulose to a carboxyl group by using 2,2,6,6-tetramethylpiperidinium oxide (TEMPO), has a large specific surface area, strong adsorption capacity, high mechanical strength, and other characteristics [11,12]. TOCNF can not only be dispersed in the aqueous phase in the form of individual fibers to form self-assembled hydrogels, but it can also be cross-linked with counterions such as Zn^2+^ (Figure 1), Ca^2+^, Al^3+^, and the carboxyl groups on the surface of TOCNF to form hydrogels. Thus, the mechanical strength and stability of the hydrogel are improved [13].

Therefore, in this study, Zn^2+^ was cross-linked with TOCNF to form hydrogels, and the effects of the TOCNF addition amount, homogenization time, and Zn^2+^ addition amount on hydrogel water absorption and porosity were explored. Furthermore, a hydrogel with good water absorption was selected for the adsorption of blueberry anthocyanins, and a hydrogel-based freshness indicator label was developed and prepared. Moreover, the feasibility of using the indicator label to detect changes in the freshness of pork mince during storage was explored, and the relevant kinetic model was established by using the Arrhenius formula to evaluate the accuracy of its indicator effect, which provided a clear reference for meat products during storage. It provides a new method for intelligent monitoring of freshness in the process of transportation and in sales.

## 2. Materials and Methods

### 2.1. Materials

TEMPO oxidizes nanocellulose (TOCNF) with carboxylic acid content of 2.0 mmol/g, fiber length of 500–1000 nm and diameter of 5–10 nm was supplied by a local retailer (Tianjin Wood Spirit Biotechnology Co., Ltd., Tianjin, China). Anhydrous zinc chloride (AR, 98%, *w*/*w*) and aqueous ammonia (AR, 25–28%, *w*/*w*) were purchased from Shanghai Aladdin Reagent Company (Shanghai, China). Blueberry anthocyanins (10%, *w*/*w*) were purchased from Shanghai Macklin Biochemical Technology Co., Ltd. (Shanghai, China). Pork was sampled from fresh hind leg meat from RT-mart (Wuhu, China). The water used in the experiment was deionized water.

### 2.2. Preparation of Nanocellulose Hydrogels

**Figure 2:**
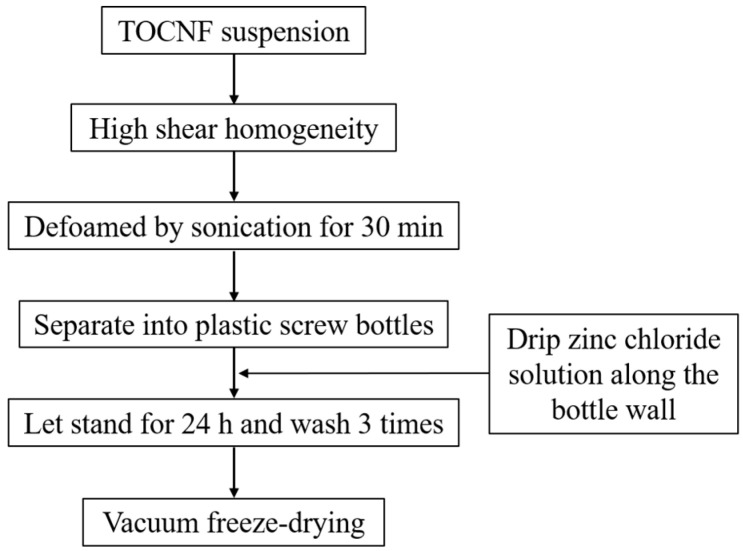
Preparation process of nanocellulose hydrogel.

The preparation of the hydrogels was performed according to the experimental method described by Lu et al. [13], as shown in Figure 2.

TOCNF suspension with a certain concentration (3%, 3.5%, 4%, 4.5% and 5% (*w*/*v*)) was homogenized for a certain period of time (3, 4, 5, 6, 7 min) by a high shear homogenizer (Fluko FA25, Shanghai Fluko Technology Development Co., Ltd., Shanghai, China), followed by 30 min ultrasonic defoaming, and then transferred quantitatively to a plastic vial with an inner diameter of 25 mm. Then, a certain volume of zinc chloride solution with a concentration of 20% (*w*/*w*) and pH = 3 was added along the inner wall of the vial to maintain the content of zinc ions in the system. The total volume of zinc chloride solution and TOCNC solution is fixed at 5 mL. Zinc chloride solution, without stirring, was directly placed at 25 °C for 24 h, and then it was washed in distilled water three times to remove excess zinc ions, freeze-drying the reserve.

#### 2.2.1. Effect of TOCNF Concentration on Water Absorption and Porosity of Hydrogels

TOCNF powder was weighed and mixed with distilled water to obtain TOCNF suspension with concentrations of 3%, 3.5%, 4%, 4.5%, and 5% (*w*/*v*), respectively. After homogenizing for 5 min (Fluko FA25, Shanghai Fluko Technology Development Co., Ltd.), the content of zinc ions in the hydrogel system was fixed at 0.25 mol/L to prepare the corresponding hydrogel, and the water absorption capacity and porosity of the hydrogel were measured.

#### 2.2.2. Effect of Homogenization Time on Water Absorption and Porosity of Hydrogels

After the TOCNF suspension with 4% concentration was homogenized for 3 min, 4 min, 5 min, 6 min and 7 min, respectively, the content of zinc ions in the hydrogel system was fixed at 0.25 mol/L to prepare the corresponding hydrogel, and the water absorption capacity and porosity of the hydrogel were measured.

#### 2.2.3. Effect of Zinc Ion Concentration on Water Absorption and Porosity of Hydrogels

After homogenizing the TOCNF suspension with 4% concentration for 5 min, the contents of zinc ions in the hydrogel system were 0.05 mol/L, 0.15 mol/L, 0.25 mol/L, 0.35 mol/L and 0.45 mol/L, respectively. The corresponding hydrogels were prepared, and the water absorption capacity and porosity of the hydrogels were measured.

### 2.3. Determination of Water Absorption Capacity of Hydrogels

The dried hydrogel was soaked in 10 mL distilled water for 24 h, and then the free water on the surface of the hydrogel was removed, and the hydrogel was weighed [14]. Water absorption rate is calculated according to Formula (1).

(1) $$Water\;absorption\;rate\left(\%\right)=\frac{W_{2}-W_{1}}{W_{1}}\times 100\%$$

where W_1_ and W_2_, respectively, represent the mass before and after water absorption of the dried hydrogel (g).

### 2.4. Determination of Hydrogel Porosity

After freeze-drying, the hydrogel was weighed, the diameter and thickness of the hydrogel were also measured, and the hydrogel was soaked in 10 mL is isopropyl alcohol solution for 24 h to fill the pores, and the mass of the hydrogel after filling the pores with isopropyl alcohol was weighed [15]. Its porosity was calculated according to Equations (2)–(4).

(2) $$P\left(\%\right)=\frac{V_{pore}}{V_{gel}+V_{pore}}\times 100\%$$

(3) $$V_{pore}=\frac{m_{wet}-m_{dry}}{\rho }\times 100\%$$

(4) $$V_{gel}=\pi R^{2}\times H$$

where V_pore_, V_gel_, m_wet_, m_dry_, *R* and H, respectively, represent the volume of hydrogel pore (cm^3^), volume of hydrogel (cm^3^), weight of hydrogel after soaking isopropyl alcohol (g), weight of freeze-dried hydrogel (g), radius of freeze-dried hydrogel (cm) and height of freeze-dried hydrogel (cm).

### 2.5. Morphological Characteristics of Hydrogels

#### 2.5.1. Scanning Electron Microscope

The dried and unground hydrogel samples were sprayed with gold, and the surface morphology of the samples was observed by SEM at 10 kV accelerating voltage [16].

#### 2.5.2. Fourier Transform Infrared Spectroscopy (FTIR)

The hydrogel samples were mixed with pure TOCNF powder and pure KBr [17]. The mixture was then ground and pressed at 100 Pa for 1 min, and the resulting transparent sheet was used for measurement. All samples were analyzed in the wavelength range of 4000~400 cm^−1^.

### 2.6. Preparation and Characterization of Indicator Label

#### 2.6.1. Response of Anthocyanins to pH

An amount of 1.0 g/L anthocyanin was obtained by dissolving 1.0 g blueberry anthocyanin in 1 L distilled water. Then, 1 mL of 1.0 g/L anthocyanin solution was added to 9 mL of solution with pH value of 1–13, stirred evenly, sealed into 25 °C incubator, and left for 30 min without light. Finally, The UV-VIS spectrum of blueberry anthocyanin solution in the pH 1–13 range was measured using a UV-VIS spectrophotometer with a scanning wavelength of 400–700 nm.

#### 2.6.2. Response of Anthocyanins to Ammonia

**Figure 3:**
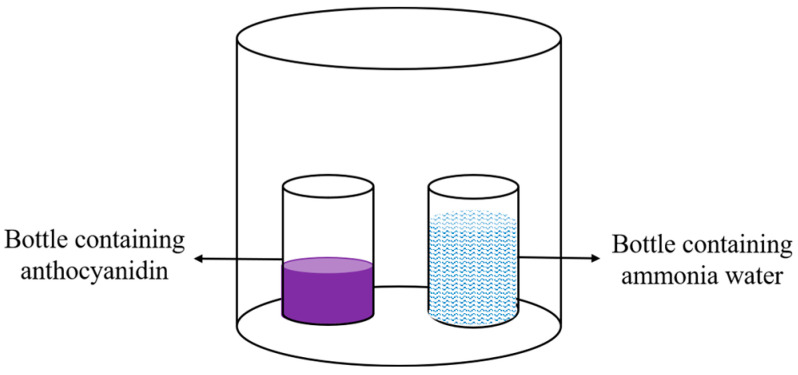
Schematic diagram of an experiment on the responsiveness of anthocyanins to ammonia.

Firstly, 10 mL of 1.0 g/L anthocyanin solution (pH = 5) was placed in a 20 mL glass bottle. Then, 20 mL of ammonia solution with a concentration of 0.028~0.28% (*w*/*w*) was placed in another glass bottle. The two glass bottles were placed in a 450 mL sealed jar as shown in Figure 3. After sealing, the anthocyanin solution was reacted at 25 °C for 3 h in the dark, and the UV–visible spectrum of the anthocyanin solution was measured using a UV spectrophotometer with a scanning wavelength of 400–700 nm [18].

#### 2.6.3. Preparation of Indicator Label

Firstly, the hydrogel was prepared according to the methods described in Section 2.2. Then, the freeze-dried hydrogel was soaked in anthocyanin solution of different concentrations (0.5–2.5 g/L) for 24 h at 25 °C, the excess water was wiped off after taking it out, and it was stored in a dark place at 4 °C.

#### 2.6.4. Response of Indicator Label to Ammonia

**Figure 4:**
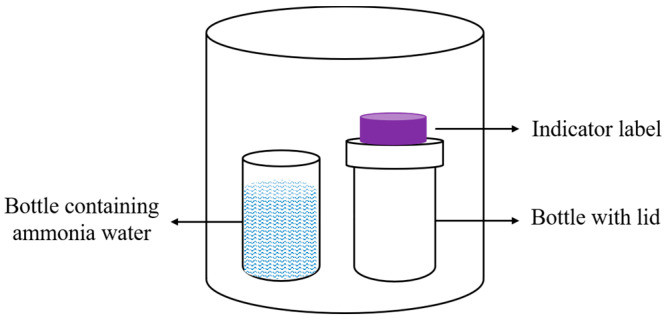
Schematic diagram of an experiment on the responsiveness of the indicator label to ammonia.

The CIE L*a*b* color coordinates of the indicator label was determined with a colorimeter (HP-C220, Hanpu Photoelectric Technology Co., Ltd., Shanghai, China). In the L*a*b* coordinates, a* represents the green–red color and b* the blue–yellow color of the samples. The L* coordinate represents lightness, where L* = 0 is completely black, and L* = 100 is completely white. The indicator label was placed over a 20 mL vial, with 20 mL of 0.14% ammonia added to another vial, and both bottles were placed in a 450 mL airtight jar as shown in Figure 4; after sealing and reacting in the dark at 25 °C for 3 h, the color change in the indicating label was measured. According to Formula (5), the total color difference in the indicating label before and after the reaction was calculated as follows:

(5) $$∆E=\sqrt{{\left({L-L}_{0}\right)}^{2}+{\left({a-a}_{0}\right)}^{2}+{{(b-b}_{0})}^{2}}$$

where *L*_0_, *a*_0_ and *b*_0_ represent the initial color value of the indicating label. *L*, *a* and *b* represent the values after the response of the indicating label, respectively [18].

#### 2.6.5. Characterization of Indicator Label

Indicator label were vacuum freeze-dried and subjected to IR and SEM analysis accorded the method as described in Section 2.5.

### 2.7. Application of Indicator Label in Detecting the Freshness of Minced Pork

#### 2.7.1. Preparation of Minced Pork

Firstly, the fat and fascia on the hind legs of fresh peeled pigs were removed and minced twice using a meat grinder. Then, 50 g of pork minced meat was placed on one side of the plastic box, and the indicator label was fixed on the other side of the plastic box. After the plastic box was sealed, it was stored at 4 °C, 14 °C, 24 °C, and 34 °C, respectively. At certain intervals, the plastic box was taken out to measure the volatile base nitrogen (TVB-N) content of pork mince, as well as the *L* (brightness), *a* (redness), and *b* (yellowness) values of the indicator label, and analysis of the total color difference at different stages was performed according to Equation (5) (Δ*E*).

#### 2.7.2. TVB-N Content Analysis

TVB-N concentration was measured according to China National Food Safety Standard methods (Determination of Total Volatile Basic Nitrogen in Food; GB/T 5009.228–2016). The results are expressed as mg of TVB-N per 100 g of pork. Finally, the freshness of minced pork was analyzed according its TVB-N content [18].

### 2.8. Statistical Analyses

Every experiment was performed in triplicate and average values with standard errors were reported. Using SPSS 25.0, statistical analysis was carried out according to analysis of variance (ANOVA) for which Duncan’s method was adopted, and graphs were produced with origin 2019b, and different lowercase letters for the same indicator was used to indicate significant differences at 5% confidence intervals (*p* < 0.05).

## 3. Results and Discussion

### 3.1. Characteristics of Hydrogel

#### 3.1.1. Water Absorption of Hydrogel

**Figure 5:**
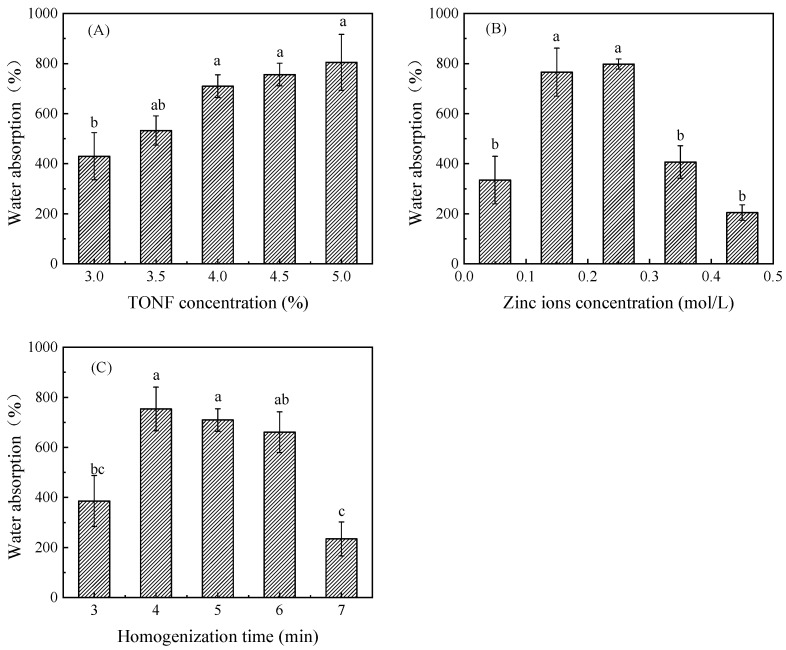
Water absorption properties of hydrogels induced by TONF (**A**) and zinc ions (**B**) and under different homogenization times (**C**). Each value represents the mean ± SD (*n* = 3). Different superscript letters (a, b, c) between columns indicate statistical differences according to Duncan’s multiple comparison test (*p* < 0.05).

The anthocyanin solution needs to be fully absorbed during the preparation of the indicator labels, so the study of the water absorption ability of the hydrogel is the prerequisite for the study of the application of hydrogels [19,20]. The influence of different factors on the water absorption of hydrogel is shown in Figure 5. Figure 5A shows the influence of the TOCNF addition amount on the water absorption of hydrogel. According to the analysis, there was a very significant effect of the TOCNF addition amount on the water absorption of hydrogel (*p* < 0.01), and with the increase in the addition amount, the water absorption gradually increased, and the high shear homogenizer used in the experiment cannot effectively homogenize the solution with the addition of nanocellulose in an amount greater than 5.0%. The maximum value was 804.81 ± 112.00% at 5.0%. Figure 5B shows the effect of zinc ion addition on the water absorption of hydrogel. According to the analysis, the zinc ion addition had a very significant effect on the water absorption of hydrogel (*p* < 0.01). With the increase in zinc ion addition, the water absorption first increased and then decreased. The highest water absorption was obtained when the content of zinc ion was controlled to 0.25 mol/L in the hydrogel system. Figure 5C shows the influence of TOCNF homogenization time on the water absorption of the hydrogel. As shown in Figure 5C, there was a very significant effect of the amount of zinc ion on the water absorption of the hydrogel (*p* < 0.01). With the increase in homogenization time, the water absorption first increased and then decreased. The highest water absorption (753.68 ± 87.23%) was obtained when the TOCNF solution was homogenized for 4 min.

A large number of hydrophilic groups in the hydrogel (such as carboxyl and hydroxyl groups contained in TOCNF) could form a hydrogen bond force with water molecules to make water molecules fixed in the hydrogel’s three-dimensional network structure, thus causing the hydrogel to rapidly absorb water and swell [21]. Therefore, the content of the carboxyl group increases with the increase in the amount of nanocellulose added, and the water absorption capacity of the hydrogel also increases. Zinc ion mainly increased the three-dimensional network structure inside the hydrogel and improved the stability of the structure by coordinating with the carboxyl group [22]. With the increase in the addition amount, the network structure of the hydrogel was improved, causing the improvement of the swelling performance of the hydrogel. However, the excessive zinc ion would also affect the interaction between the internal hydrophilic group carboxyl group and water and reduce the water absorption performance [22,23], making the water absorption first decline and then increase with the increase in zinc ion. During the homogenization process of TOCNF solution, cellulose was mechanically wrapped and folded to form a three-dimensional network structure with low stability. As the homogenization time increases, the effect of wrapping and folding became excessive, which also affected the subsequent addition of zinc ions that could not fully penetrate into the interior of TOCNF for coordination and binding, thereby affecting water absorption. As the homogenization time increases, the water absorption first increased and then decreased.

#### 3.1.2. The Porosity of Hydrogel

**Figure 6:**
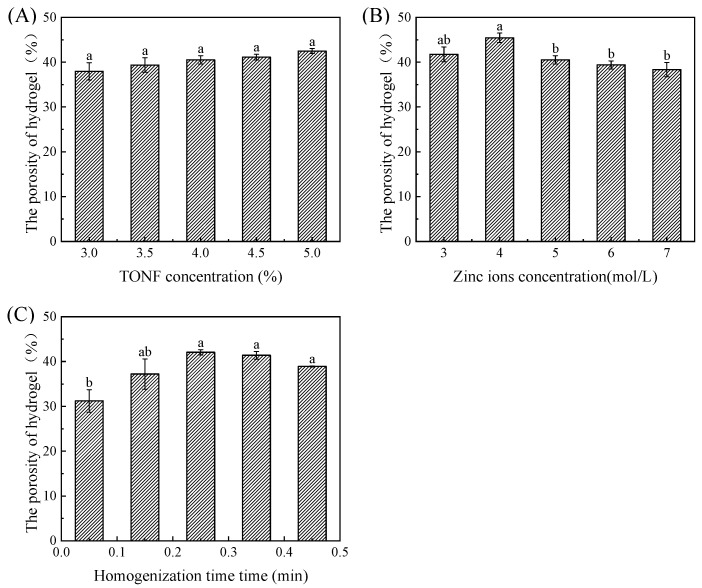
The porosity of hydrogels induced by TONF (**A**) and zinc ions (**B**) and under different homogenization times (**C**). Each value represents the mean ± SD (*n* = 3). Different superscript letters (a, b) between columns indicate statistical differences according to Duncan’s multiple comparison test (*p* < 0.05).

Porosity is an important physical property of hydrogels. High porosity means that the material has a large specific surface area and high adsorption capacity, which is more conducive to the subsequent adsorption of the anthocyanin solution to prepare indicator labels [8]. The influence of different factors on the porosity of hydrogel is shown in Figure 6. As shown in the figure, the addition of TOCNF has no significant effect on the porosity of the hydrogel (*p* > 0.05). Figure 6B shows the effect of zinc ion addition on the porosity of hydrogel. As shown in Figure 6B, the amount of zinc ion added has a very significant impact on the porosity of the hydrogel (*p* < 0.01). With the increase in zinc ion addition, the porosity first increased and then decreased. The highest porosity (42.07 ± 0.59%) was achieved when the zinc ion addition was 0.25 mol/L in the hydrogel system. Figure 6C shows the effect of the TOCNF homogenization time on the porosity of hydrogel. As shown in Figure 6B, the homogenization time has a very significant effect on the porosity of the hydrogel (*p* < 0.01). As the homogenization time increases, the porosity shows a trend of first increasing and then decreasing, with the highest porosity (45.44 ± 1.05%) at 4 min of TOCNF suspension homogenization.

Under the mechanical force of the high shear homogenizer, the TOCNF solution folds and tangles between TOCNF fibers to form a three-dimensional structure. The addition of zinc ions enhanced the interaction between carboxyl groups to produce the three-dimensional structure more stable. However, excessive zinc ions induced the reduction in particle size of the hydrogel, resulting in an increase in pore density and porosity [13,22]. However, a long time of homogenization treatment leads to strong folding and entanglement effect of cellulose molecules, which leads to the inability of zinc ions to penetrate into the gel, thus reducing the stability, and the pore structure is also easily destroyed, which reduces the porosity.

### 3.2. Preparation of Indicator Labels

#### 3.2.1. Response of Anthocyanins to pH

**Figure 7:**
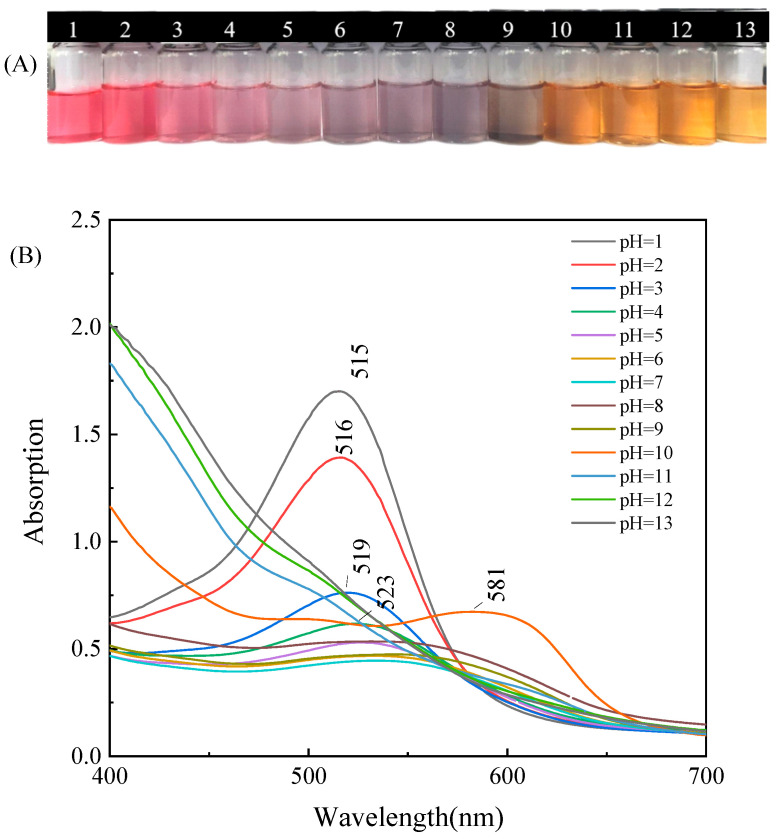
Color (**A**) and visible spectral absorption (**B**) characteristics of blueberry anthocyanin solution at pH 1–13.

**Figure 8:**
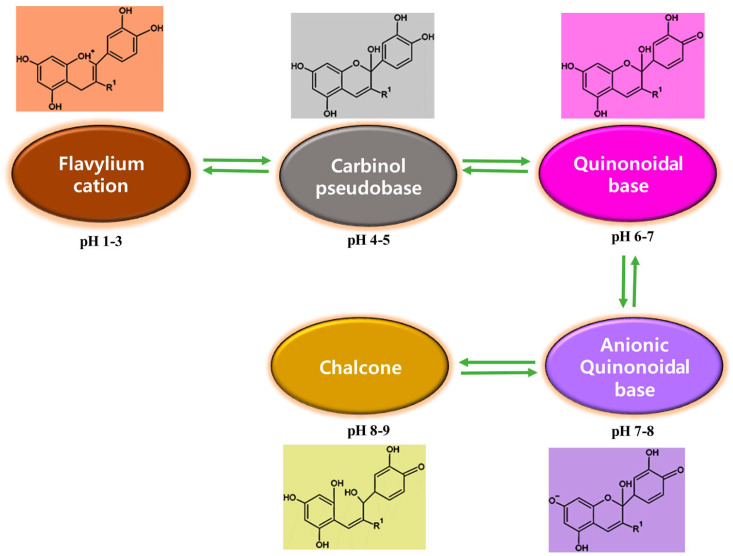
Molecular structure and color changes in blueberry anthocyanins at different pH.

Anthocyanins exhibit different colors under different pH conditions. As shown in Figure 7A, within the pH range of 1–13, the color changes in blueberry anthocyanins are in the order of red, light pink, purple, blue purple, gray purple, and yellow brown. The color change in the solution is due to the mutual transformation of the high-molecular-weight conjugated system of anthocyanin molecules under different acid and base conditions. The four molecular structures of anthocyanins with different colors, namely, xanthate cation (red), methanol pseudobase (colorless), quinone base (blue), and chalcone (colorless), have different degrees of mutual transformation due to the increase and decrease in H^+^ and OH^−^ ion content. Studies have demonstrated that pH value has a significant impact on the stability and color changes in anthocyanins during storage. The anthocyanin solution appeared red/pink in strong acidic conditions (pH < 2), purple/blue in a relatively neutral medium (pH 6–7), and green/yellow under alkaline conditions, which were the colors with greatest dominance (Figure 8) [24]. In addition, anthocyanin 3-glucoside achieved the highest stability at pH 8–9, while other glycosides achieved the highest stability at pH 5–7.

Figure 7B shows the UV–visible spectrum of anthocyanin solution. Generally, the greater the absorbance in the visible spectrum, the deeper the color development of the solution. As shown in Figure 7B, within the pH range of 1–5, as the pH increased, the maximum absorption wavelength of the solution shifted from 515 nm to 523 nm, and the absorbance value decreased significantly. When the pH of the solution increased to 10, the maximum absorption wavelength was located near 581 nm. In the pH range of 11–13, the maximum absorption peak of the solution disappeared, and the shape of the spectrum remained unchanged. That is because, under strong alkaline conditions, the stability of anthocyanins was poor, and the central ring of the anthocyanin molecule decomposed to form chalcone. The absorbance and wavelength of blueberry anthocyanins were constantly changing due to the structural transformation of anthocyanins caused by pH changes [24].

#### 3.2.2. Response of Anthocyanins to Ammonia

**Figure 9:**
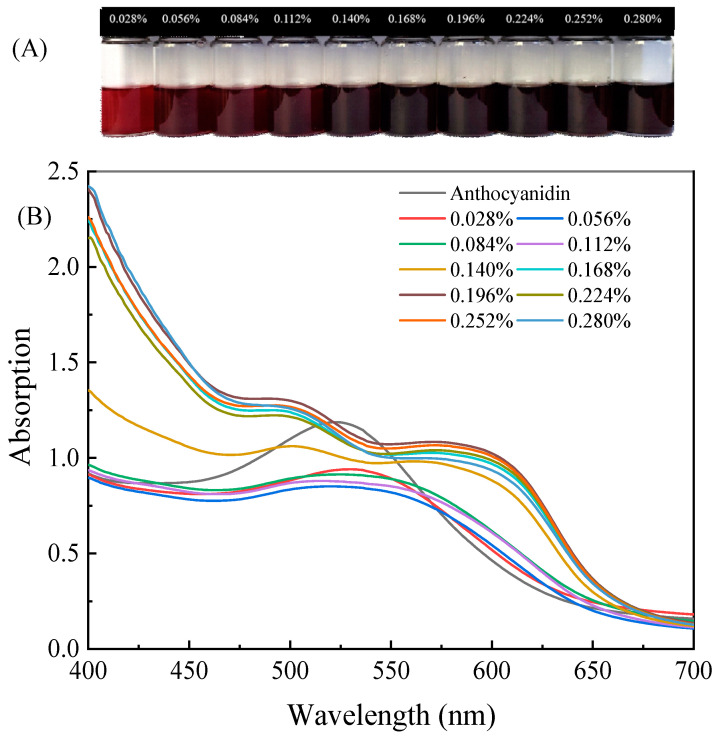
Color (**A**) and visible spectral absorption (**B**) characteristics of blueberry anthocyanin solution for different concentrations of ammonia.

When ammonia is dissolved in water, it forms ammonium ions and hydroxide ions, leading to a change in the pH of the solution and causing a change in the color of the indicator. The color response of the anthocyanin indicator solution to different concentrations of ammonia is shown in Figure 9A. As the concentration of ammonia solution increases from 0.028% to 0.14%, the color of the solution gradually changed from bright red to purple red and then to purple. Then, as the concentration of ammonia increased to 0.28%, the color of anthocyanin remained purple. Moreover, it was observed with the naked eye that only changes in color depth were observed in anthocyanin solutions with an ammonia concentration greater than 0.14%.

The UV–visible absorption spectrum corresponding to the reaction between anthocyanins and ammonia is shown in Figure 9B. It was observed that an absorption peak appeared near 530 nm in a 1 g/L blueberry anthocyanin solution that was not in contact with ammonia. As the ammonia concentration increased, the absorption peak gradually shifted to the right, and the intensity of the absorption peak gradually decreased. This was also due to the solubility of ammonia in water causing an increase in pH, and the yellow salt cations gradually transformed into colorless methanol pseudobases and blue quinone-type bases. However, when the ammonia concentration increased to 0.14%, the absorption peak disappeared. It indicated that the pH of the solution was sufficient to open the central ring of anthocyanins, and the color of the anthocyanin solution also changed to deep purple [18].

As the concentration of ammonia water continued to increase, there was no significant change in the spectral pattern. Although the absorption intensity increased at 0.168% ammonia concentration, there was no significant change in absorption intensity as the ammonia concentration continued to increase. The results indicate that anthocyanin solution had a relatively high sensitivity to ammonia [18,20], and the color of anthocyanin solution varied with the change in ammonia concentration in the range of 0.028% to 0.14%. Therefore, in the subsequent preparation of indicator labels, it was considered best to use 0.14% ammonia concentration for ammonia response experiments to ensure significant color changes and reduce the waste of ammonia in the experiment.

#### 3.2.3. The Color Difference in Color Indicator Labels

**Figure 10:**
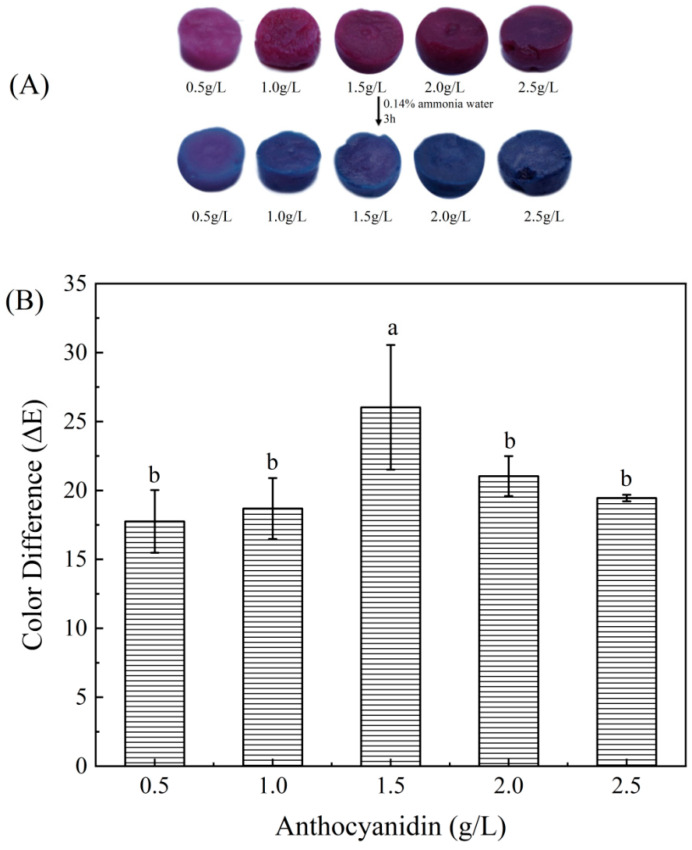
Color (**A**) and total color difference change (**B**) of indicator labels obtained by soaking anthocyanin solutions of different concentrations. Each value represents the mean ± SD (*n* = 3). Different superscript letters (a, b) between columns indicate statistical differences according to Duncan’s multiple comparison test (*p* < 0.05).

Under the action of microorganisms and enzymes, volatile nitrogen-containing compounds such as ammonia, trimethylamine, and dimethylamine are produced during the spoilage process of meat. Therefore, ammonia gas can be used to simulate the volatile nitrogen-containing compounds generated during the process of meat spoilage, in order to explore the response sensitivity of indicator labels obtained by soaking in different concentrations of anthocyanin solutions [20]. Figure 10A shows the color changes in the indicator labels on ammonia gas obtained by soaking different concentrations of anthocyanin solutions. It can be observed that the color of the indicator labels changed from purple red to blue after 3 h of exposure to ammonia gas. As the soaking concentration increases, the starting purple red color of the indicator labels becomes darker, and the blue color after the reaction ends also becomes darker. Figure 10B shows the total color difference changes in different indicator labels before and after exposure to ammonia gas. As shown in the figure, as the concentration of the anthocyanin solution used to prepare the indicator label increased, the total color difference in anthocyanins showed a tendency of first increasing and then decreasing. As the content of anthocyanins that reacted with ammonia increased, the indicator labels soaked in 1.5 g/L anthocyanin concentration has the maximum total color difference before and after contact with ammonia (19.718 ± 0.434). However, when the anthocyanin concentration continuously increased, the color change which was caused by a pH change induced by ammonia reaching saturation. Moreover, the deep red color inherent in anthocyanins also affected the color rendering effect, leading to a decreasing tendency in total color difference. Therefore, the indicator labels obtained by soaking 1.5 g/L anthocyanin solution was selected for experiments relating to the freshness detection of pork mince.

#### 3.2.4. Characterization of Indicator Labels

##### Infrared Spectrum of Indicator Labels

**Figure 11:**
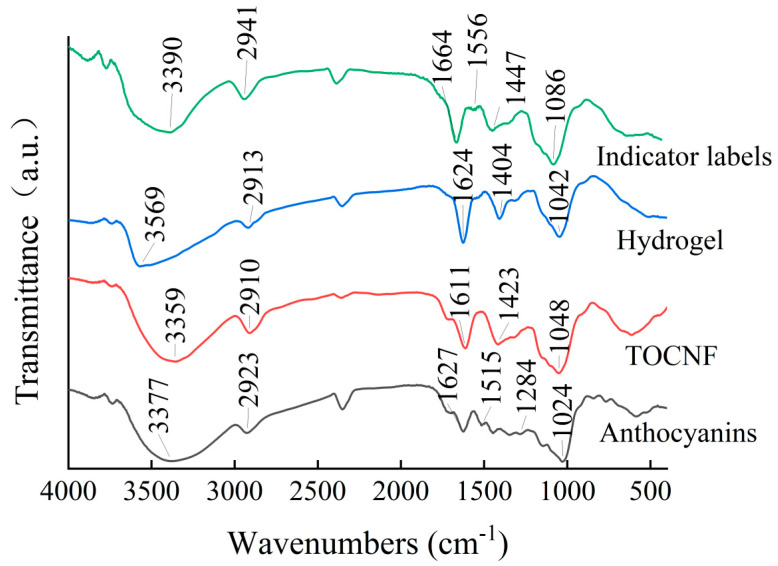
Infrared spectrum of hydrogel and indicator label.

The infrared spectra of TOCNF, anthocyanins, hydrogels, and indicator labels are shown in Figure 11. As shown in the figure, there is a strong and wide absorption peak near 3377 cm^−1^ for blueberry anthocyanins and TOCNF, mainly due to the presence of more hydroxyl groups in both anthocyanins and TOCNF. Moreover, the peak showed a significant red shift in the hydrogel prepared by TOCNF, which might be due to the increase in the number of hydrogen bonds during the preparation of the hydrogel. Compared with the single TOCNF, the infrared spectrum of the indicator labels prepared by adsorption of anthocyanins showed a blue shift. This is mainly because the addition of anthocyanins increased the hydroxyl content in the sample, which also indicated that anthocyanins were successfully adsorbed into the hydrogel [8,19]. At 1611 cm^−1^ and 1423 cm^−1^, the characteristic peaks of TOCNF were observed, namely, the symmetric and asymmetric stretching vibration peaks of -COOH. The characteristic peaks of anthocyanins at 1627 cm^−1^ and 1515 cm^−1^ were caused by the stretching vibration of C=C on the aromatic ring skeleton of anthocyanins, and the absorption peak at 1284 cm^−1^ was caused by the stretching vibration of the pyran ring in its structure. The absorption peak at 1015cm^−1^ might be caused by the bending vibration of C-H on the aromatic ring. The blue shift in the COOH vibration peak in the hydrogel sample may be caused by the coordination bond between zinc ion and carboxyl group. Moreover, the carboxyl vibration peak in the indicator label showed a red shift, and the absorption peak at 1556 cm^−1^ indicated that anthocyanins were adsorbed on the hydrogel and affected the original coordination bond. According to FT-IR analysis, blueberry anthocyanins can attach well to hydrogels and have good compatibility with TOCNF. The performance of indicator labels is mainly determined by intermolecular forces, and the chemical composition of each substrate is not affected [9].

##### Scanning Electron Microscope for Indicating Labels

The SEM image can be used to determine the dispersion and compatibility of various components within the indicator labels. It was found by scanning electron microscope that the cellulose fibers inside the freeze-dried hydrogel were coiled and wound, with an obvious pore structure, but the diameters of the holes were different, with diameters ranging from 60 to 100 μm. Therefore, the hydrogel prepared by shear homogenization and the cross-linking of zinc ion and TOCNF had good water absorption and porosity, and it has a certain application value.

**Figure 12:**
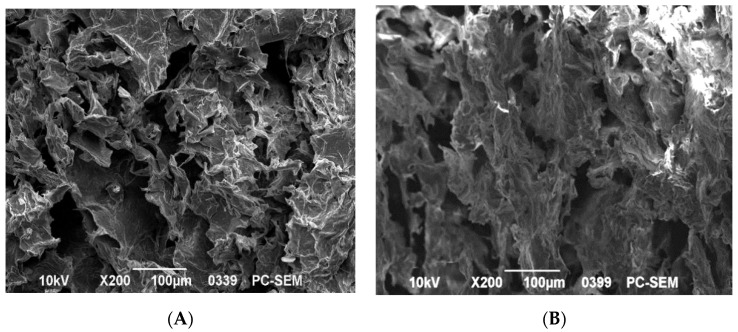
SEM image of hydrogel (**A**) and indicator label (**B**).

Compared with the hydrogel shown in Figure 12A, the indicator labels with blueberry anthocyanin in Figure 12B showed a more compact and smooth surface, indicating that the blueberry anthocyanin and TOCNF hydrogel had good compatibility. In addition, the pore size of the hydrogel prepared with anthocyanin and TOCNF were smaller than that of the hydrogel. After the anthocyanins were adsorbed, the pore size of the hydrogel is 45 μm, and there were some small particles of anthocyanins attached to cellulose aggregates. This might be because the adsorption of anthocyanins successfully attached to the hydrogel filled the original pore structure. Moreover, the phenolic hydroxyl groups in blueberry anthocyanin molecules formed intermolecular hydrogen bonds with the hydroxyl groups in nanocellulose [8,25], reducing the phenomenon of polymer chain molecular entanglement and hydrogen bonding between cellulose molecules. Eventually, the mechanical properties of the hydrogel were affected, making the internal structure more uniform.

### 3.3. Application of Indicator Labels in Detecting the Freshness of Pork Mince

#### 3.3.1. Freshness of Pork Mince and Indicator Labels Color

**Figure 13:**
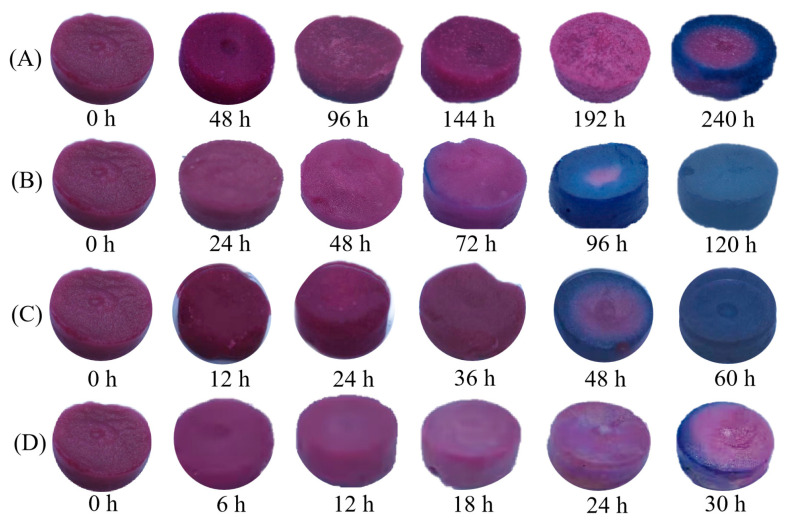
Color change in indicator labels (**A**–**D**) under 4, 14, 24 and 34 °C storage condition.

**Figure 14:**
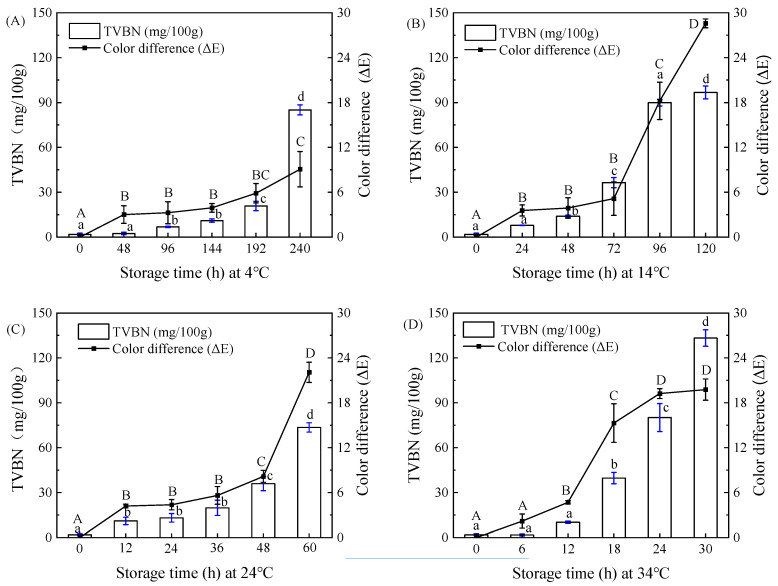
TVBN and total color difference in indicator labels (**A**–**D**) under 4, 14, 24 and 34 °C storage condition. Each value represents the mean ± SD (*n* = 3). Different superscript lowercase letters (a, b, c, d) between columns and capital letters (A, B, C, D) between points of curve indicate statistical differences according to Duncan’s multiple comparison test (*p* < 0.05).

The changes in the indicator label color, TVBN, and total color difference in pork mince stored at different storage temperatures (4–34 °C) are shown in Figure 13. As shown in Figure 14A–D, with the extension of storage time, the TVBN value and Δ*E* was constantly increasing, with an initial value of 1.90 mg/100 g for TVBN. In accordance with the GB2707-2016 ‘Hygienic Standard for Fresh (Frozen) Animal Meat’, the TVBN value for fresh meat should be less than 15 mg/100 g, and the indicator label should be purple red at this condition. As shown in Figure 13A–D, with the extension of storage time, it was observed that the color of the indicator label changes from purple red to blue purple at different temperatures, and there were certain differences in the time and degree of color change. According to the change in total color difference between TVBN and indicator label in Figure 14A–D, it was inferred that this might be due to differences in TVBN values greater than 15 mg/100 g at different temperatures. The TVBN value at 4 °C at 192 h was 20.85 mg/100 g, which exceeded the freshness standard for pork mince. And, at this point, the minced meat Δ*E* was 5.88 ± 1.30, and the color was observed by the naked eye to change from initial red to light purple. Subsequently, at 240 h, the TVBN value of pork mince was 85.09 mg/100 g, Δ*E* was 9.09 ± 2.37, and the edge of the label was seen by the naked eye as blue purple. Similarly, the TVBN value for 72 h at a storage temperature of 14 °C was 36.46 mg/100 g, Δ*E* was 5.15 ± 2.13, and the edge of the indicator label could be visually observed to appear blue purple. With the extension of storage time, the blue range of the indicator label expands until it completely turns blue purple at 120 h, at which point the TVBN value is 96.72 mg/100 g. The TVBN value at 24 °C for the 36 h was 19.78 mg/100 g, and Δ*E* was 5.62 ± 1.89, indicating that the purple color of the label deepens, and as the storage time prolongs, the label gradually turned blue until it completely turned blue purple at 60 h. At this time, the TVBN value was 73.53 mg/100 g.

The TVBN value at 34 °C at 18 h was 39.68 mg/100 g, and Δ*E* was 15.29 ± 2.58, indicating that the surface of the label turns blue. With the extension of storage time, the blue color of the label deepened. The final TVBN value at 60 h was 133.25 mg/100 g, Δ*E* was 19.76 ± 1.42, and a clear blue purple color was observed on the indicator labels. There were certain differences in the types and metabolites of microorganisms at different temperatures, which resulted in differences in the range and color depth of the blue indicator labels at different temperatures. However, when the TVBN value exceeded 15 mg/100 g, the indicator labels for detecting the freshness of pork mince at different temperatures showed a change from purple red to blue purple. Some freshness indicators have been designed based on the volatile compounds released by microbial spoilage, thus resulting in the pH increase inside the package headspace. For example, an on-package indicator label was developed by Chen et al. [4] for monitoring lean pork freshness on the basis of the presence of TVB-N in the package. It was found that the lower initial pH is, the slower color change is. This colorimetric freshness indicator provides three different colors for illustrating freshness (red), medium freshness (goldenrod), and spoilage (green), thus enhancing guarantee of pork safety. In contrast, visual pH-sensing films containing curcumin and anthocyanins were developed as on-package indicator labels for monitoring fish freshness in real time [26]. This SPVA/glycerol film incorporated with curcumin and anthocyanins at a ratio of 2:8 (*v*/*v*) could provide three different colors, which were assigned to the sign of freshness, medium freshness, and spoilage for packaged fish. Furthermore, efficient prediction dynamic modeling of the freshness and freshness indicator labels of pork mince is needed to explore the correlation between the indicator labels and the quality of pork mince at different temperatures.

#### 3.3.2. Dynamics Analysis of TVBN and Indicator Labels Color in Pork Mince

Mathematical modeling is an effective method for analyzing and predicting various biological and chemical reactions that cause changes in food quality. The Arrhenius equation is a commonly used empirical formula to represent chemical reaction rates and temperature changes, which can explore the impact of temperature on the rate of change in experimental objects. For example, the response change in the indicator labels can be modeled as a function of the response time (t) and the reaction rate constant (k) of color change. After the K value of the indicator label is determined, the activation energy (Ea) can be calculated using the Arrhenius equation. Based on the dynamic model established for pork mince and freshness indicator labels, the activation energy of pork and indicator label changes could be obtained. However, it is generally believed that the difference in activation energy between the appropriate freshness indicator label and the food should be less than 25 kJ/mol. Establishing a kinetic model can also accurately reflect the impact of temperature changes around the food on the food and the indicator label, and it can provide predictive factors for the food quality change process during the shelf life.

Analyzing the TVBN content in meat is beneficial for assessing food quality. Therefore, the changes in TVBN content of pork mince stored at 4 °C, 14 °C, 24 °C, and 34 °C at different time points were used to analyze the quality change trend of pork mince and determine its kinetic parameters. For fresh pork quality changes, the first order kinetic equation should be used to analyze the relationship between TVBN content of pork mince and time.

**Figure 15:**
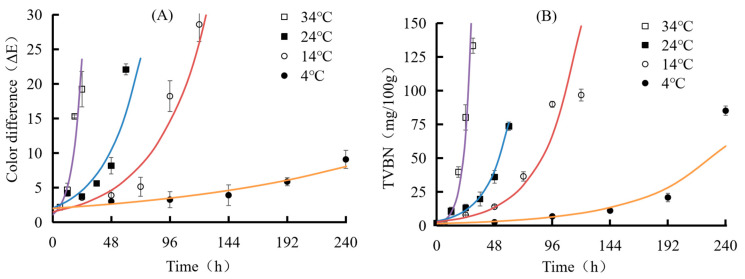
Changes in pork TVBN (**A**) and color of indicator label (**B**) at different temperatures.

The trend and kinetic parameters of TVBN changes in pork mince at different temperatures are shown in Figure 15 and Table 1 and Table 2. According to Figure 15, as the storage time prolongs, the color difference and TVBN of indicator labels at different storage temperatures showed a gradual upward trend, and the rate of change also varied at different temperatures. The first-order kinetic equation is suitable for analyzing the changes in food quality of fresh meat. Therefore, it is necessary to select an exponential function for linear fitting of the TVBN content changes in pork mince and the color difference in the indicator label. The response function and change rate of the TVBN and indicator color difference values of pork mince are shown in Table 1 and Table 2. It was found that with the increase in temperature, the change rate constants of TVBN and total color difference were both increasing. According to Arrhenius equation, 1/RT corresponding to different temperatures was taken as the abscissa (R is the molar gas constant, also known as the ideal gas constant, with the value of 8.3144 J/mol·K; T is the thermodynamic temperature, with the unit of K), and the logarithm ln k of the change rate constant corresponding to the temperature was taken as the ordinate to draw and perform linear fitting, as shown in Figure 15. The relationship between the label change rate and temperature obtained by linear fitting is ln k (total color difference) = −68337/RT + 24.595, and the linear fitting R^2^ = 0.9587. According to this equation, the activation energy indicating the total color difference in the label is 68.337 kJ/mol. Significantly, a means to correlate color changes to freshness of packaged foods must be established. Sun et al. [27] reported a back propagation artificial neural network model which was established using the color of the films as the input and the TVB-N content as the output. It was found that neural network model well predicted the freshness of packaged pork patties and fish balls based on color change in the films, with R^2^ of 0.94–0.98.

**Figure 16:**
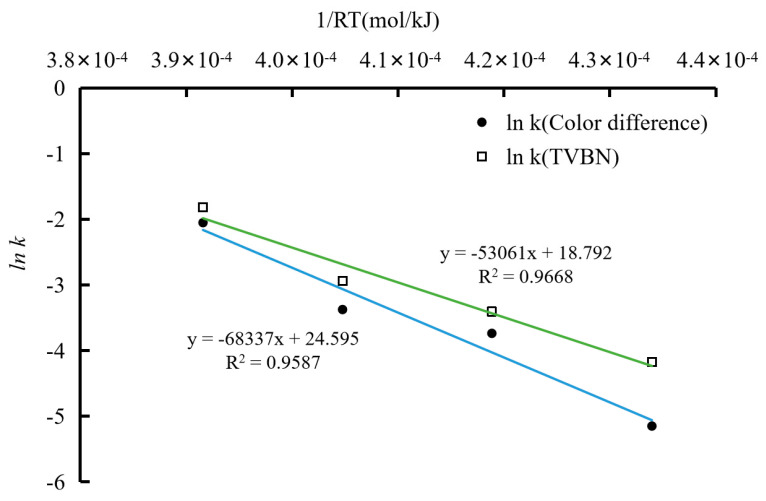
The ln k and 1/RT curves of pork TVBN and total color difference in indicator labels at different temperatures.

The Arrhenius equation for TVBN and indicator color difference response factor can be obtained by fitting the equation of 1/RT and ln k, as follows (Figure 16):

ln k (TVBN) = −53,061/RT + 18.792(6)

ln k (Color difference)= −68,337/RT + 24.595(7)

According to the equation, the activation energies of TVBN and total color difference are 53,061 J/mol and 68,337 J/mol, respectively, with a difference of less than 25 kJ/mol. According to Taoukis’ viewpoint, a good freshness indicator label that can achieve real-time monitoring of food quality should meet the activation energy of the food (i.e., TVBN content of pork mince) Ea1 and the activation energy of the indicator (total color difference before and after the indicator label) Ea2, with a difference of less than 25 kJ/mol. At four different temperatures, the color change in indicator labels was consistent with TVBN content of ground pork exceeded the spoilage threshold (greater than 15 mg/100 g). Therefore, the anthocyanin indicator labels could maintain good responsiveness at different storage temperatures. It is suitable for detecting the content of volatile nitrogen-containing odor gas produced in the process of pork ground corruption in various scenarios and for realizing real-time monitoring of meat freshness.

## 4. Conclusions

The hydrogel prepared by using zinc ion and TEMPO-oxidized nanocellulose (TOCNF) had good water absorption and a stable three-dimensional network structure. The indicator label made from the hydrogel-adsorbed blueberry anthocyanin showed obvious color change in the experiment of using ammonia gas to simulate the corruption of pork surimi, and a corresponding maximum color difference was observed before and after the hydrogel indicator labels soaked in 1.5 g/L anthocyanin solution. Applying this indicator label to the detection of pork mince freshness, it was found that when pork mince spoils, the labels changed from purple red to blue purple. Moreover, based on the Arrhenius formula, a dynamic model was established for the color difference value and TVBN change in the indicator label, and it was found that the indicator labels maintained similar color changes according to the freshness of pork mince at different temperatures. In conclusion, blueberry anthocyanin is an ideal intelligent food packaging indicator, and the hydrogel indicator label prepared by anthocyanin and nanocellulose has good application prospects in the detection of pork mince freshness.

## Figures and Tables

**Table 1:** Response function and change rate of pork TVBN at different temperatures.

Storage Temperature (°C)	Change Trend of TVBN	R^2^	The Rate Constants	ln k
34	y = 1.3777e^0.162x^	0.9471	0.162	−1.820158944
24	y = 3.1861e^0.0529x^	0.9125	0.0529	−2.93935194
14	y = 2.7466e^0.0332x^	0.9559	0.0332	−3.405205403
4	y = 1.4663e^0.0154x^	0.9621	0.0154	−4.17338777

**Table 2:** Response function and change rate of color of indicator labels at different temperatures.

Storage Temperature (°C)	Change trend of ∆*E*	R^2^	The Rate Constants (∆*E*)	ln k (Color Difference)
34	y = 1.0812e^0.1284x^	0.8609	0.1284	−2.0526
24	y = 2.0219e^0.0342x^	0.8695	0.0342	−3.37553
14	y = 1.4846e^0.0238x^	0.9607	0.0238	−3.73807
4	y = 1.9988e^0.0058x^	0.9447	0.0058	−5.1499

## Data Availability

The datasets generated for this study are available on request to the corresponding author.
